# *FASN* Gene Methylation is Associated with Fatty Acid Synthase Expression and Clinical-genomic Features of Prostate Cancer

**DOI:** 10.1158/2767-9764.CRC-23-0248

**Published:** 2024-01-18

**Authors:** Oluwademilade Dairo, Lia DePaula Oliveira, Ethan Schaffer, Thiago Vidotto, Adrianna A. Mendes, Jiayun Lu, Sophie Vo Huynh, Jessica Hicks, Adam G. Sowalsky, Angelo M. De Marzo, Corrine E. Joshu, Brian Hanratty, Karen S. Sfanos, William B. Isaacs, Michael C. Haffner, Tamara L. Lotan

**Affiliations:** 1 Department of Pathology, Johns Hopkins School of Medicine, Baltimore, Maryland.; 2 Department of Epidemiology, Johns Hopkins Bloomberg School of Public Health, Baltimore, Maryland.; 3 Laboratory of Genitourinary Cancer Pathogenesis, NCI, Bethesda, Maryland.; 4 Divisions of Human Biology and Clinical Research, Fred Hutchinson Cancer Center, Seattle, Washington.; 5 Department of Urology, Johns Hopkins School of Medicine, Baltimore, Maryland.; 6 Department of Oncology, Johns Hopkins School of Medicine, Baltimore, Maryland.

## Abstract

Fatty acid synthase (FASN) catalyzes the synthesis of long-chain saturated fatty acids and is overexpressed during prostatic tumorigenesis, where it is the therapeutic target in several ongoing trials. However, the mechanism of FASN upregulation in prostate cancer remains unclear. Here, we examine *FASN* gene CpG methylation pattern by InfiniumEPIC profiling and whole-genome bisulfite sequencing across multiple racially diverse primary and metastatic prostate cancer cohorts, comparing with FASN protein expression as measured by digitally quantified IHC assay and reverse phase protein array analysis or *FASN* gene expression. We demonstrate that the *FASN* gene body is hypomethylated and overexpressed in primary prostate tumors compared with benign tissue, and *FASN* gene methylation is significantly inversely correlated with FASN protein or gene expression in both primary and metastatic prostate cancer. Primary prostate tumors with *ERG* gene rearrangement have increased FASN expression and we find evidence of *FASN* hypomethylation in this context. FASN expression is also significantly increased in prostate tumors from carriers of the germline *HOXB13* G84E mutation compared with matched controls, consistent with a report that HOXB13 may contribute to epigenetic regulation of FASN *in vitro*. However, in contrast to previous studies, we find no significant association of FASN expression or methylation with self-identified race in models that include *ERG* status across two independent primary tumor cohorts. Taken together, these data support a potential epigenetic mechanism for FASN regulation in the prostate which may be relevant for selecting patients responsive to FASN inhibitors.

**Significance:**

Here, we leverage multiple independent primary and metastatic prostate cancer cohorts to demonstrate that *FASN* gene body methylation is highly inversely correlated with *FASN* gene and protein expression. This finding may shed light on epigenetic mechanisms of FASN regulation in prostate cancer and provides a potentially useful biomarker for selecting patients in future trials of FASN inhibitors.

## Introduction

Fatty acid synthase (FASN) is a key metabolic enzyme that catalyzes *de novo* synthesis of long-chain fatty acids (1, 2). In normal prostate epithelium, as in most normal tissues, FASN expression is relatively low due to diet-obtained endogenous free fatty acid (3). However, significant upregulation of FASN occurs during prostatic tumorigenesis, with the highest levels expressed in metastatic disease (4, 5). Increased FASN expression is associated with disease progression and adverse oncologic outcomes, particularly in men with increased body mass index (1, 6–9). Supporting its potential role as an oncogene, FASN overexpression in the prostates of transgenic mice is sufficient to cause prostatic intraepithelial neoplasia (PIN; ref. 10). Pharmacologic inhibition of FASN inhibits growth of preclinical models of castration-resistant prostate cancer (CRPC), and downregulates androgen signaling, suggesting that FASN may be a potential therapeutic target in the disease (9, 11).

Despite its central role, the precise mechanisms of FASN upregulation during prostatic tumorigenesis have remained elusive, but are critical to understand as more clinical trials of FASN inhibitors are initiated. The *FASN* gene is recurrently amplified in up to one quarter of primary and metastatic prostate cancers, and gene amplification correlates with FASN protein expression to some extent (7). However, *FASN* gene amplification is reportedly not present in PIN (7), despite documented increased FASN protein expression in these premalignant prostate lesions compared with benign glands (5). Consistent with these data suggesting that additional mechanisms beyond gene amplification may modulate FASN expression in prostate cancer, transcriptional regulation of FASN during prostatic tumorigenesis has also been demonstrated. *FASN* gene expression is higher in androgen receptor (AR)-positive LNCaP cells than AR-negative DU145 or PC3 cells, and early studies demonstrated that androgen treatment *in vitro* increases *FASN* mRNA and activity levels in cells that express AR (12). More recent chromatin immunoprecipitation sequencing studies have confirmed that AR binds to the *FASN* promoter region in primary prostate tumors, with particular enrichment in tumors from self-identified Black (BL) men (13), and the increased expression of FASN in *ERG*-rearranged tumors may also be consistent with a role for AR in FASN regulation (14). However, AR is expressed and androgen signaling is active even in benign prostate epithelial cells, where FASN levels remain very low, suggesting additional mechanisms are likely involved.

More recently, there has been some suggestion that epigenetic regulation of FASN may be critical in prostate cancer. For example, HOXB13, a homeobox family transcription factor essential for prostatic development that regulates the AR cistrome, suppresses FASN levels via recruitment of histone deacetylases in model systems, independent of AR (15). However, the role of epigenetic regulation of *FASN* has not been extensively studied. In the current study, we investigate *FASN* gene CpG methylation pattern in human prostate cancer samples. We demonstrate that FASN protein is upregulated and the *FASN* gene is concomitantly globally hypomethylated in primary prostate tumors compared with normal tissue across multiple independent datasets. Furthermore, in both primary tumors and CRPC, FASN expression levels are significantly correlated with global *FASN* gene methylation. Finally, increased FASN expression is present in tumors from germline carriers of the *HOXB13* G84E mutation, supporting a role for HOXB13 in FASN regulation.

## Materials and Methods

### Patient and Tissue Samples for Immunostaining

This study was conducted under a waiver of consent from the Johns Hopkins Institutional Review Board in accordance with the US Common Rule. To analyze tumor FASN immunostaining, we utilized three primary tumor cohorts: The first was a previously described radical prostatectomy cohort from 1995 to 2010 of 177 self-identified BL and 194 self-identified White (WH) men, matched by Grade Group from Johns Hopkins (JHU cohort; refs. 16–20). We recently published Infinium EPIC methylation profiling data on a subset of this cohort (21) as described below, and samples were also arrayed on tissue microarray (TMA) for immunostaining studies as described previously (16). The second cohort was also from JHU but included in the Prostate Cancer Biorepository Network (PCBN cohort) and comprised 57 WH and 58 BL men with radical prostatectomies occurring between 2014 and 2016, also matched for Grade Group and selected to overrepresent higher Grade Groups, and arrayed on a TMA (22–26). The third cohort was a previously described (27) cohort of 93 heterozygous germline carriers of *HOXB13* G84E who underwent radical prostatectomy for prostate cancer between 1985 and 2011 and matched by race, age, and tumor grade to 92 germline *HOXB13* wild-type (WT) controls, arrayed on a TMA. Patients undergoing radical prostatectomy did not have prior prostatic radiotherapy or chemotherapy administered for prostate cancer.

### Patient and Tissue Samples for Genome-wide Methylation Profiling, Reverse Phase Protein Analysis, RNA Sequencing, and RNA Microarray Profiling

Three previously published cohorts of primary prostate tumors with methylation array profiling were leveraged for this study. The first consisted of 145 tumor samples from BL men and 145 tumor samples from WH men, who comprised a subset of the JHU cohort with matched TMAs described above. In addition, a subset of 111 of these patients had RNA microarray profiling which has been previously published on the Decipher platform (19). DNA samples isolated from formalin-fixed paraffin-embedded tumors were analyzed using the Infinium EPIC methylation array platform as described recently, and compared with 30 benign prostate tissues from a subset of the WH and BL men (21). The second cohort was the previously published The Cancer Genome Atlas (TCGA) prostatic adenocarcinoma cohort, where Infinium 450K methylation array profiling data were available from tumors from 502 men, with matched benign samples from 50 men (28). From among these samples, RNA sequencing (RNA-seq) data were available on 537, and reverse phase protein analysis (RPPA) data available on 350. The third cohort was an unpublished public dataset of radical prostatectomy samples and matched benign tissues profiled by Infinium EPIC methylation profiling (NCI cohort, GSE183040). The NCI cohort was comprised of 84 tumor samples and 142 benign samples (blood and matched benign prostate tissue) with available RNA-seq data. Methylation data were available for 58 tumor samples and for 58 benign-adjacent prostate tissues.

Finally, we also leveraged the Stand Up to Cancer (SU2C) West Coast Dream Team (WCDT) metastatic CRPC cohort for which whole-genome bisulfite sequencing (WGBS; ref. 29) and RNA-seq data were published previously (30). For this cohort, we utilized data from a subset of 48 cases where paired WGBS and expression data were available and tumor purity was estimated at >60%.

### FASN and p63 Immunostaining

IHC for FASN was conducted on 4-µm-thick sections from the JHU and PCBN TMAs utilizing a rabbit monoclonal antibody for FASN (Cell Signaling Technology, catalog no. 3180, RRID: AB_2100796) and the Ventana Benchmark immunostaining system (Ventana/Roche; RRID:SCR_021254). To enable antigen retrieval, slides were incubated with CC1 retrieval solution at 100°C for 32 minutes, and the primary antibody was incubated for 40 minutes at a dilution of 1:100. Detection and counterstain reagents used were the OptiView DAB kit (Roche, 760-700; RRID: AB_2833075), hematoxylin and bluing reagents, respectively. The IHC assay was validated using two positive NCI-60 control cell lines (SK-MEL-5, RRID: CVCL_0527 and T47D, RRID: CVCL_0I95) with high *FASN* RNA expression based on RNA-seq and another NCI-60 cell line with low FASN RNA expression (RXF-393, RRID: CVCL_1673; ref. 31; Supplementary Fig. S1).

FASN-stained TMA slides were scanned at 20x magnification on the NanoZoomer HT Scanner (RRID:SCR_021658). After scanning, each TMA slide was de-cover-slipped, double stained with p63 [mouse monoclonal (4A4), Abcam, #ab735, RRID: AB_305870,1:100, OptiView DAB kit] to distinguish basal cells identifying benign glands and rescanned.

### FASN Image Analysis

Image analysis was performed on QuPath v0.2.2, RRID:SCR_018257, an open source software for digital image analysis (32) where histoscore (H-score) was used to quantify IHC staining intensity. To annotate all epithelial cells within a given TMA spot in QuPath, a pixel classification with a DAB threshold of 0.05 was performed. Benign gland annotations were then manually deleted by visual assessment of p63 immunostaining to detect presence of basal cells. Then, an intensity classification in the remaining tumor glands was performed with the DAB OD Max threshold, which was set to three different thresholds [weak (1+), moderate (2+), and strong (3+)] of staining intensity to correlate with visual analysis (Supplementary Fig. S2A). The analysis algorithm was executed to obtain each tumor core's H-score and the obtained datapoints were exported to analyze for average and maximum H-scores per case. The digital scoring data were visually examined by a pathologist for all scored cores from each TMA. For benign gland analysis performed on a single TMA for comparison with tumor gland analysis, steps were identical to above, except that the tumor gland annotations were manually deleted by visual assessment of absence of p63-positive basal cells and benign gland annotations were carried forward for subsequent H-score analysis. In all cases, the H-score was evaluated on the double-stained (FASN/p63) slide to enable exclusion of benign glands with p63-positive nuclei, after confirming that there was high correlation between the analysis performed on the double-stained slide and that performed on the single stained slide (Supplementary Fig. S2B).

### *FASN* Methylation Analysis by Infinium Arrays

Methylation pipelines were conducted according to our previously published study (21). Briefly, for JHU and NCI (GSE183019) cohorts, raw Infinium EPIC array methylation data were processed and normalized via SWAN (Subset quantile Within-Array Normalization) method using *minfi* in R. Individual beta values for CpGs were then obtained for all 850,000 methylation sites. DNA methylation beta values are continuous variables between 0 and 1, representing the proportion of methylation for a given CpG site, calculated as the ratio of the intensity of the methylated bead type to the combined methylated and unmethylated bead intensity for the specific probe. Overall annotation for CpGs was used to select all 56 probes located up to 1,500 bp upstream or within the *FASN* gene body. Mean beta value for *FASN* was obtained by calculating the average beta for all 56 probes per sample. For TCGA and NCI cohorts, mean *FASN* beta values were obtained by calculating the average beta across 55 and 56 probes, respectively, as TCGA methylation data was obtained from Illumina 450k methylation arrays containing only 55 probes.

### *FASN* Gene and Protein Expression and ERG Status Assessment by Gene Expression

*FASN* gene expression analysis was performed for JHU, TCGA, and NCI cohorts. For the JHU cohort, gene expression analyses were performed as previously described using Human Exon 1.0 ST microarrays (Decipher Biosciences; ref. 19). For TCGA, normalized counts for tumor and benign samples were downloaded from the BROAD Institute TCGA Firehose platform (https://gdac.broadinstitute.org/). For the NCI cohort, transcript per million (TPM)-normalized gene expression was obtained from the Gene Expression Omnibus (GEO) database (accession number GSE183019). *ERG* fusion status was assessed from *ERG* gene expression for both TCGA and NCI cohorts. Normalized gene expression was used as input for an expectation-maximization (EM) algorithm to calculate cut-off points based on two normal distributions. *ERG* was then dichotomized into ERG+ and ERG− independently for both cohorts. *FASN* protein expression analysis on TCGA cohort was performed by employing RPPA data obtained from the cBioPortal database.

### *FASN* Methylation Analysis by WGBS

WGBS for the WCDT cohort was reported previously and data were processed as described previously (29). Methylation data were visualized by first extracting coordinate and percent methylated information from the methylation call format into bedGraph format and then converting to bigWigs using bedGraphToBigWig (33). BigWig files were then viewed in the integrative genomics viewer using default setting (34).

### Data Availability

Scanned FASN IHC whole slide images and processed data will be made available by request to the corresponding author. *FASN* methylation data are previously published and available in the GEO repository (GSE221219; ref. 21). For the JHU cohort, gene expression microarray data were previously published and available on GEO (GSE153352; ref. 19). For the NCI cohort, methylation data are available on GEO (GSE183019). For the WCDT cohort, methylation data were published previously (29) and available on dbGAP (phs001648). Data from TCGA cohort (28) are available through cBioportal.

## Results

### FASN is Upregulated and Hypomethylated in Primary Prostate Tumors Compared with Benign Tissue

**FIGURE 1:**
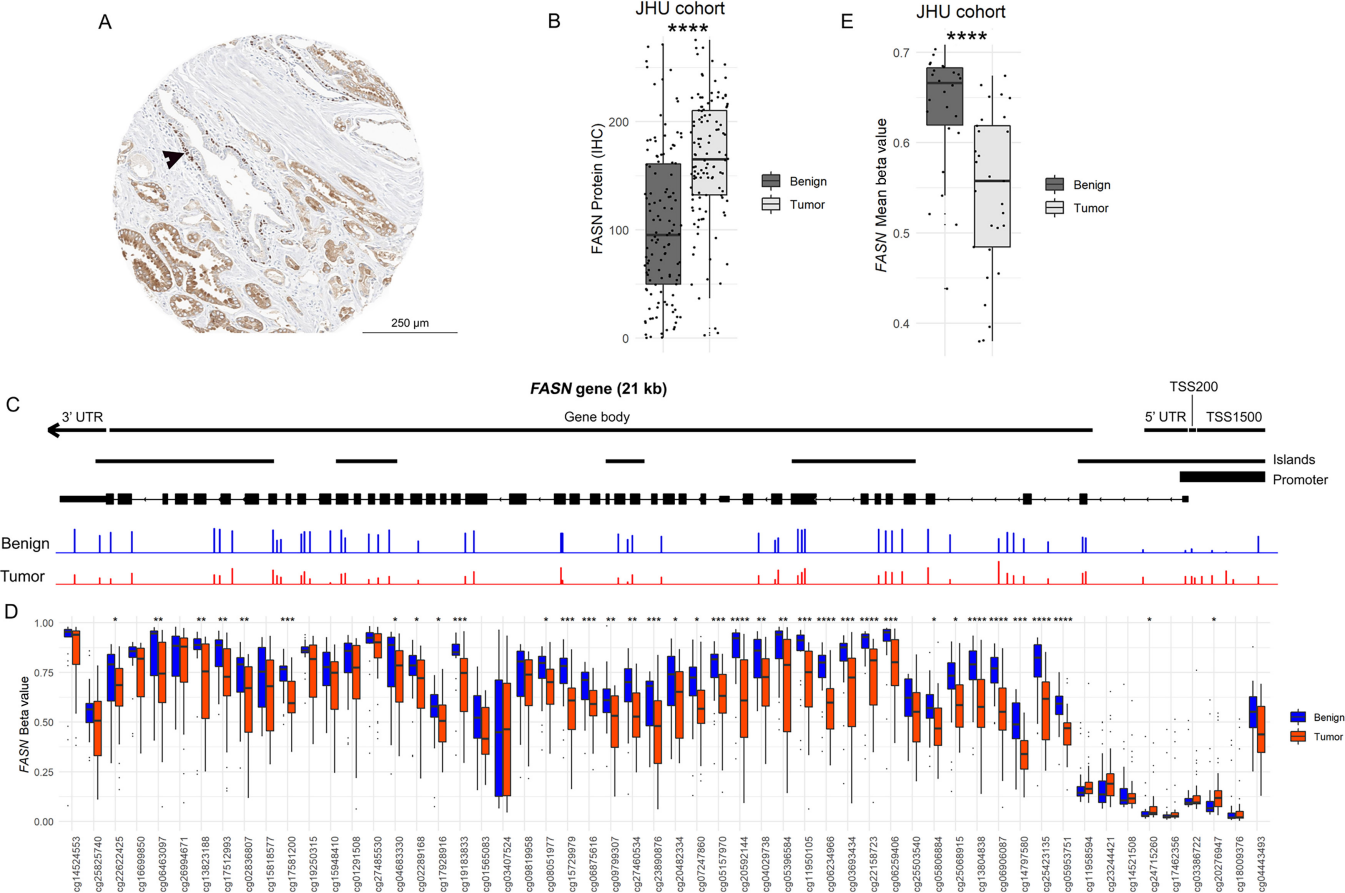
FASN protein is upregulated and *FASN* gene is hypomethylated in tumor compared with benign tissue in JHU primary tumor cohort. **A,** Dual FASN and p63 IHC assay in representative primary prostate tumor from JHU cohort (scale bar = 250 µm). FASN and p63 are both labeled in brown. Benign glands with nuclear p63 labeling in basal cells (arrow) are then manually excluded from analysis and FASN scoring is performed on tumor glands only. See Supplementary Fig. S2. **B,** Quantified FASN protein expression by immunostaining in benign prostate glands versus tumor glands from JHU cohort; each point represents the mean H-score from a single prostatectomy sample, analyzed in a matched analysis. **C,** Representative plots of beta values (range: 0–1) by *FASN* probe on InfiniumEPIC for a representative paired benign (blue) and tumor (red) sample set from the JHU cohort. TSS: transcription start site; UTR: untranslated region. **D,***FASN* gene methylation beta values by *FASN* probe for benign tissue versus tumor tissue in JHU cohort. **E,** Mean *FASN* gene methylation probe beta values for benign tissue versus tumor tissue in JHU cohort. Each point represents the mean beta value from a single prostatectomy sample analyzed in a matched analysis (*, *P* < 0.05; **, *P* < 0.01; ***, *P* < 0.001; ****, *P* < 0.0001).

To assess epithelial FASN protein expression across a racially diverse JHU primary tumor cohort, we digitally quantified a validated FASN IHC assay (Supplementary Fig. S1) in benign glands and primary prostate tumor epithelial cells (Supplementary Fig. S2). As described in previous studies, FASN protein was upregulated in tumor glands compared with adjacent benign epithelial cells (Fig. 1A) and this difference was statistically significant across 115 normal-tumor pairs (*P* < 0.0001; Fig. 1B). Examination of *FASN* gene expression by bulk RNA-seq in TCGA cohort (Supplementary Fig. S3) or another publicly available primary tumor dataset from the NCI (Supplementary Fig. S4) demonstrated similar results at the RNA level with increases in *FASN* expression in tumors relative to unpaired benign samples.

To query whether hypomethylation in the *FASN* gene might be associated with upregulation of *FASN* RNA and protein in tumor, we examined previously published CpG methylation array data across all three cohorts described above. On this platform, there are 56 (Infinium EPIC) or 55 (Infinium 450K) probes capturing unique CpG sites across *FASN* (Fig. 1C), including those upstream from the transcription start site (TSS) and those in the gene body or 3′ untranslated region. While mean beta values reflecting methylation status were uniformly low for probes in the region of the TSS, higher methylation was seen globally across the gene body, where the majority of these CpG sites were significantly hypomethylated in tumor samples compared with the matched benign tissue from the same case across 30 normal-tumor pairs from the JHU cohort (Fig. 1D). When methylation across the *FASN* gene body was summarized as the mean beta value across all 56 probes for each sample, tumor samples were significantly hypomethylated compared with matched benign tissue in a matched analysis (*P* < 0.0001; Fig. 1E). Similar differences were observed in TCGA (*P* < 0.0001; Supplementary Fig. S3) and NCI (*P* < 0.01; Supplementary Fig. S4) datasets in unmatched analyses.

### FASN Protein Expression and FASN Gene Methylation are Inversely Correlated in Tumor Tissue

**FIGURE 2:**
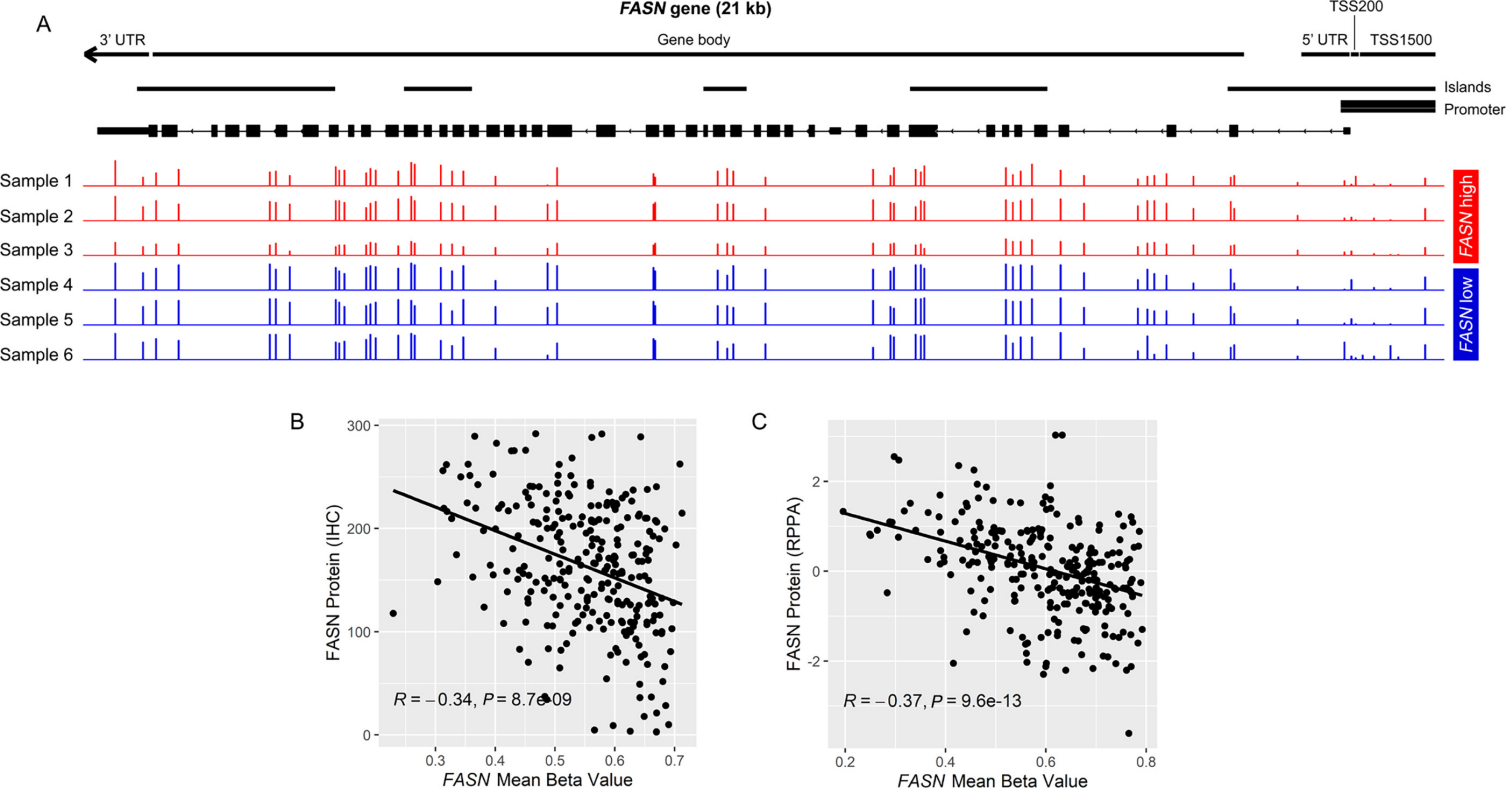
FASN protein expression in tumor glands is inversely correlated with *FASN* gene methylation in tumor tissue in primary prostate tumors. **A,** Representative plots of beta values (range: 0–1) by *FASN* probe on InfiniumEPIC for three tumors among the lowest (blue, H-score range from 3–4) and highest (red, H-score range from 289–292) mean FASN protein expression in the JHU cohort. Mean *FASN* beta values are negatively correlated with FASN protein levels by immunostaining for tumors in JHU cohort (**B**) and with FASN protein levels by RPPA in TCGA cohort (**C**). Each point in B and C represents an individual tumor.

Given that *FASN* gene body hypomethylation was associated with its upregulation in tumor compared with benign tissue, we next queried whether *FASN* methylation level was correlated with FASN gene or protein expression among primary tumors. In the JHU cohort, cases with the highest digitally quantified FASN protein expression by immunostaining showed lower methylation across nearly all probes compared with cases with the lowest FASN protein expression (Fig. 2A). Accordingly, the inverse correlation between FASN protein expression and global *FASN* gene methylation as captured by mean beta value was highly significant (*R* = −0.34; *P* = 8.7 × 10^−9^; Fig. 2B), with comparable correlations seen for each individual probe (Supplementary Table S1). A similar inverse correlation between protein expression and global methylation was seen in TCGA cohort, where FASN protein levels were measured by RPPA (*R* = −0.37; *P* = 9.6 × 10^−13^; Fig. 2C), with comparable correlations across individual probes (Supplementary Table S1). A significant inverse correlation was also seen for *FASN* gene expression versus *FASN* mean beta value among the subset of JHU samples with available mRNA microarray data (*R* = −0.57; *P* = 2 × 10^−8^; Supplementary Fig. S5), as well as in TCGA cohort (*R* = −0.55; *P* < 2.2 × 10^−16^; Supplementary Fig. S5) and the NCI cohort (*R* = −0.29; *P* = 0.027; Supplementary Fig. S5).

**FIGURE 3:**
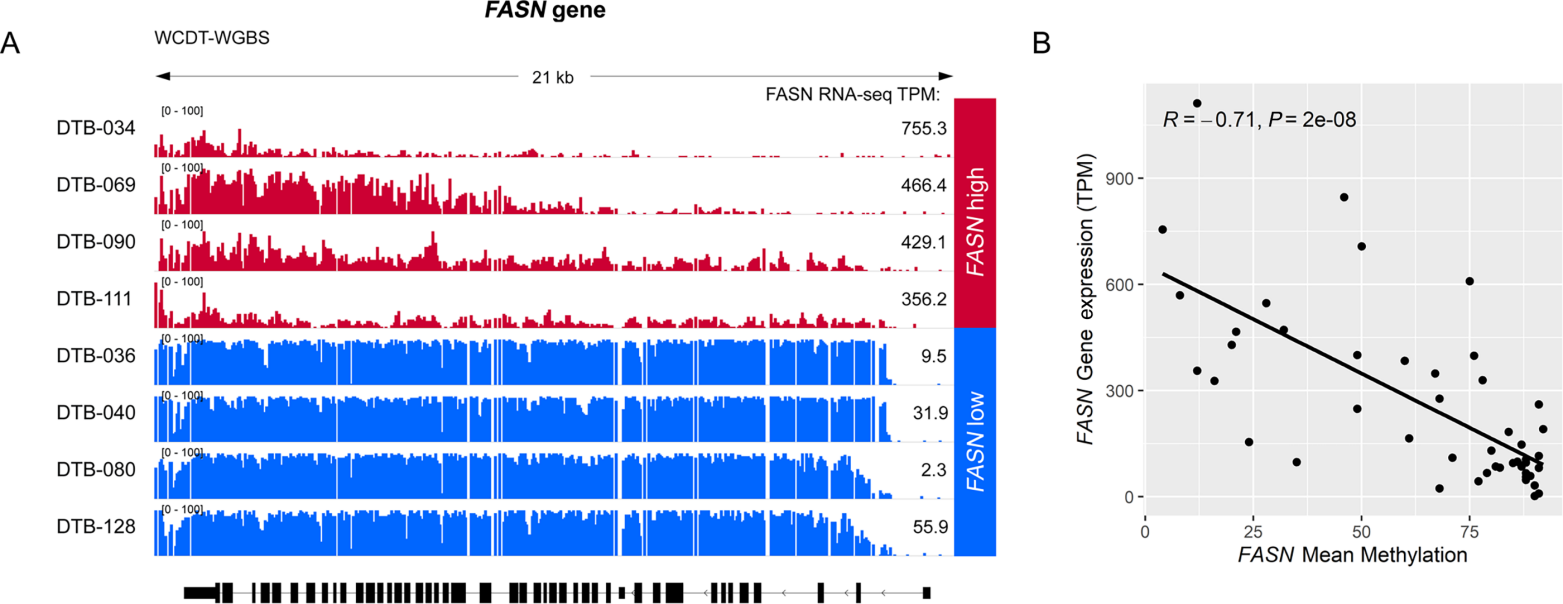
FASN protein expression in tumor glands is inversely correlated with *FASN* gene methylation in tumor tissue in metastatic CRPC cohort. **A,** Representative plots of CpG methylation level in *FASN* by WGBS with corresponding FASN mRNA expression (TPM) for three representative tumors with low (blue) and high (red) FASN gene expression in the SU2C WCDT cohort. Mean *FASN* CpG methylation level is negatively correlated with FASN mRNA expression in the SU2C WCDT samples; each point represents an individual tumor in **B**.

To test whether the inverse correlation between global *FASN* gene methylation and expression observed in primary tumors was generalizable to metastatic tumors and other methylation assays, we examined WGBS previously published for the SU2C WCDT metastatic prostate tumor samples (29). Cases with the lowest *FASN* expression showed uniformly high methylation across 1178 CpG sites in the *FASN* locus, compared with cases with the highest *FASN* gene expression (Fig. 3A). Accordingly, there was a significant inverse correlation between *FASN* gene expression and mean methylation level across all CpG sites (*R* = −0.71; *P* = 2 × 10^−8^; Fig. 3B). In contrast, other gene loci that are highly expressed in prostate tumor cells, such as *TMPRSS2* and *KLK3* showed very different (and expected) methylation patterns in the same dataset (Supplementary Fig. S6), excluding the possibility of an artifact in the WGBS. Taken together, these data suggest that methylation could be a mechanism regulating FASN expression levels in primary tumors and metastatic prostate cancer.

### Association of FASN Expression and Methylation with ERG Status in Primary Prostate Cancer

**FIGURE 4:**
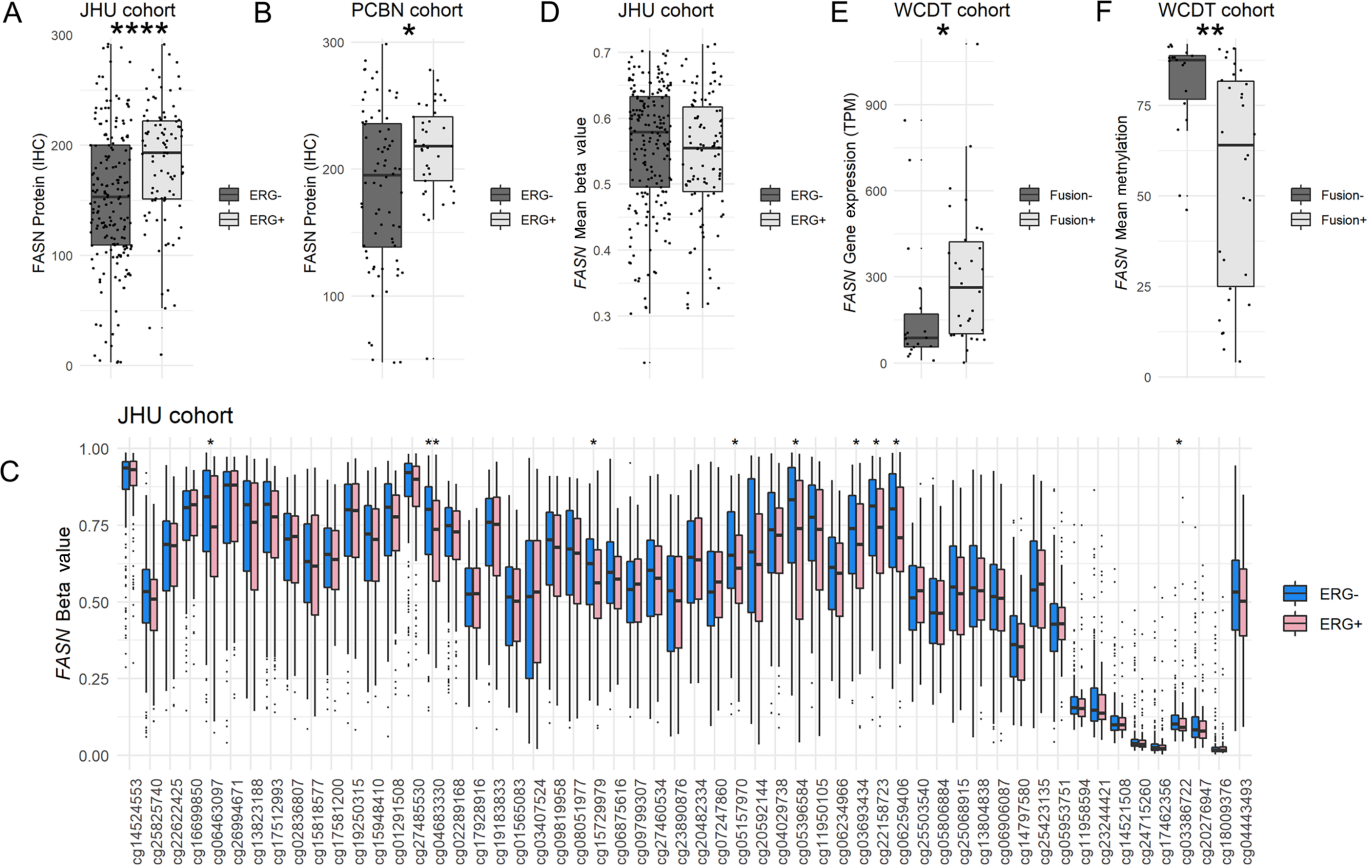
FASN protein expression, and to a lesser extent *FASN* gene methylation, is associated with ERG status in primary tumor cohort. **A,** FASN protein expression by immunostaining is higher in ERG+ compared with ERG− cases in the JHU primary tumor cohort; each point represents an individual tumor. **B,** FASN protein expression by immunostaining is also increased in ERG+ compared with ERG− cases from the PCBN cohort; each point represents an individual tumor. **C,***FASN* probe methylation varies significantly by ERG status for several probes. **D,***FASN* gene methylation mean beta value is not significantly different by ERG status in JHU cohort; each point represents an individual tumor. **E,***FASN* gene expression is significantly higher in ERG fusion positive compared with ERG fusion negative tumors in the WCDT cohort; each point represents an individual tumor. **F,***FASN* gene mean methylation is significantly lower in ERG fusion positive compared with ERG fusion negative tumors in the WCDT cohort; each point represents an individual tumor (*, *P* < 0.05; **, *P* < 0.01; ***, *P* < 0.001; ****, *P* < 0.0001).

FASN protein expression has previously been shown to be increased in prostate tumors harboring underlying *ERG* gene rearrangements compared with those without *ERG* rearrangements (14). Using ERG expression by IHC as a genetically validated proxy for *ERG* rearrangement status (35), we were able to corroborate this finding in the JHU primary tumor cohort (*P* < 0.0001; Fig. 4A) with a similar trend in a separate independent primary tumor cohort from the PCBN (*P* = 0.05; Fig. 4B). Using a previously described excitation-maximization algorithm for *ERG* gene expression level to dichotomize cases by ERG status in TCGA cohort (36), *FASN* gene expression level by RNA-seq (though not FASN protein level by RPPA) was also significantly higher in ERG-positive compared with ERG-negative cases (*P* < 0.0001; Supplementary Fig. S7). A similar finding for *FASN* gene expression was replicated in the NCI cohort (Supplementary Fig. S8).

FASN is an androgen-regulated gene in prostate cancer (12) and ERG has been previously described as a pioneer-like factor for AR (37), raising the question of whether AR activity differences in ERG-positive and -negative tumors may underlie the association of FASN with ERG status. As expected, measures of AR activity were significantly positively correlated with FASN gene expression in both the JHU [*R* = 0.42, *P* < 0.0001 for previously published androgen receptor activity (ARA) score (38)] and TCGA [*R* = 0.18, *P* = 0.0036 for previously published AR score (28, 36)] cohorts. However, this finding does not explain the association of higher FASN expression with ERG status because AR activity scores were similar for ERG-positive versus -negative cases in the JHU cohort (*P* = 0.64 for ARA score) and actually significantly lower for ERG-positive compared with negative cases in TCGA cohort (*P* < 0.0001 for AR score). Taken together, these data do not support the hypothesis that differences in AR activity underlie differences in FASN expression by ERG status.

To determine whether differences in FASN expression by *ERG* rearrangement status might be associated with underlying differences in *FASN* methylation, we examined probe-level and global *FASN* methylation in both the JHU and TCGA datasets. In the JHU cohort, a few individual probes in the *FASN* gene body showed significantly increased methylation in ERG-negative compared with ERG-positive cases (*P* < 0.05, Fig. 4C), and the median across the tumor samples’ mean beta values was numerically, though not statistically significantly, higher for ERG-negative compared with ERG-positive cases (Fig. 4D). Interestingly, a similar and more significant trend was seen in TCGA cohort, both at the individual probe level in the 5′ gene body (Supplementary Fig. S7) and by mean beta value (*P* < 0.01; Supplementary Fig. S7). These findings were replicated in the WCDT metastatic tumor cohort, where *FASN* gene expression was significantly higher in *ETS* fusion positive versus negative cases (*P* < 0.05; Fig. 4E) and mean *FASN* gene methylation by WGBS was concomitantly significantly lower in *ETS* fusion positive versus negative cases (*P* < 0.01; Fig. 4F).

### Association of *FASN* Expression with *HOXB13* G84E Carrier Status

**FIGURE 5:**
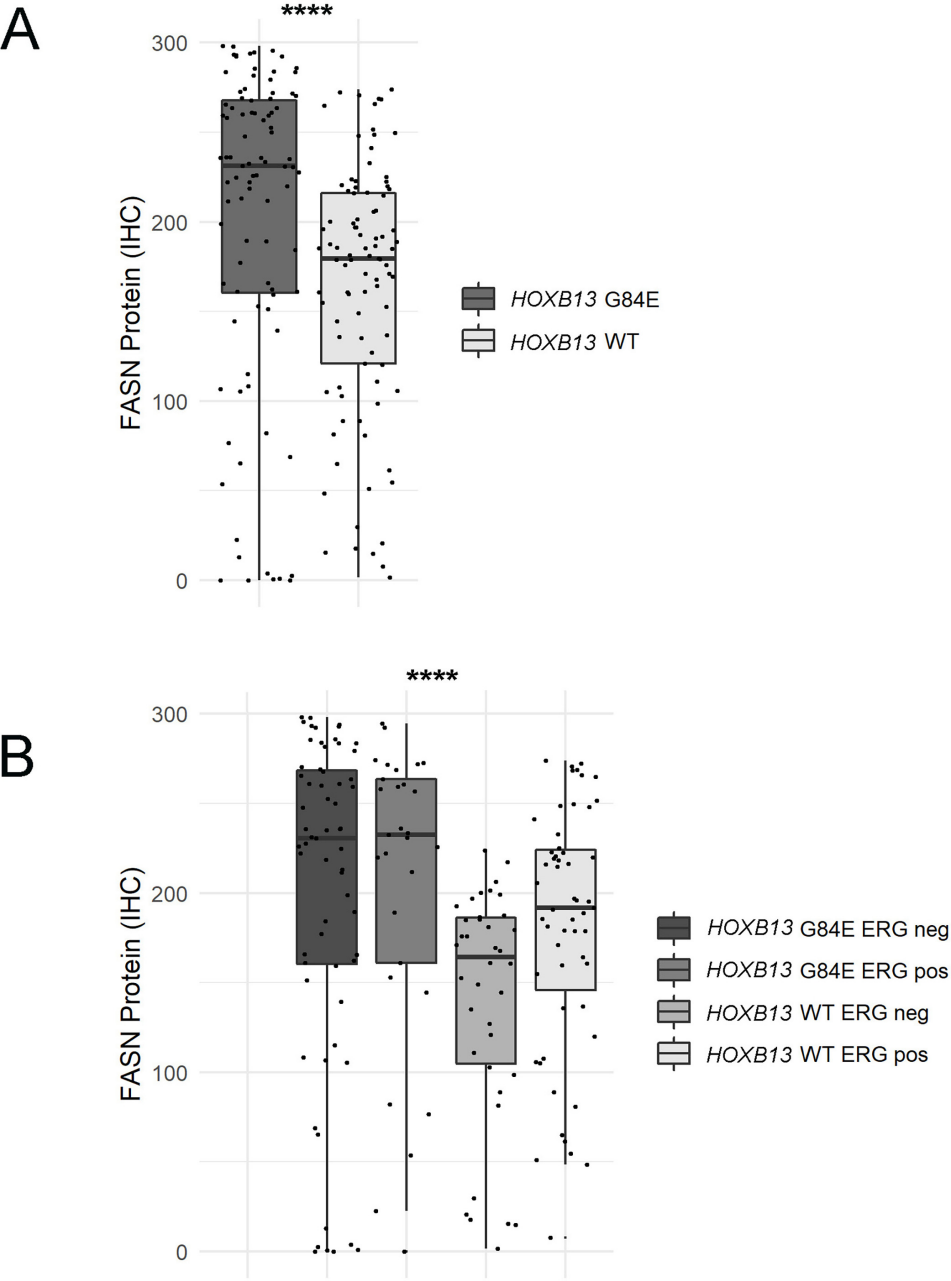
FASN protein expression is increased in prostate tumors from germline *HOXB13* G84E carriers compared with matched WT controls. **A,** FASN protein expression by immunostaining is higher in *HOXB13* G84E carriers than matched *HOXB13* WT controls. **B,** ERG status is not associated with FASN protein expression among *HOXB13* G84E carriers, though higher expression is seen among ERG-positive compared with ERG-negative tumors from *HOXB13* WT controls (****, *P* < 0.0001).

The only prior study of epigenetic regulation of FASN has suggested that HOXB13 may function to repress FASN expression in prostate cancer cells, while the *HOXB13* G84E mutation may lead to FASN derepression and increased expression (15). To test this directly in human tissues, we quantified FASN immunostaining using a previously described group of 92 carriers of *HOXB13* G84E matched to 93 *HOXB13* WT controls (27). Strikingly, FASN protein expression was significantly higher among the *HOXB13* G84E carrier tumors compared with controls (Fig. 5A) and this was independent of ERG status in this group (Fig. 5B), though FASN expression was higher in ERG-positive compared with ERG-negative tumors from *HOXB13* WT controls as expected.

### Association of FASN Expression and Methylation with Clinical-pathologic Characteristics and Outcome in Primary Prostate Cancer

Finally, we examined whether FASN protein expression and/or methylation was correlated with clinical or pathologic variables in the JHU or PCBN cohorts. There was no significant correlation between quantified FASN protein expression and patient age (*r* = −0.10, *P* = 0.05 for JHU or *r* = 0.01, *P* = 0.9 for PCBN) or preoperative PSA (*r* = −0.06, *P* = 0.3 for JHU or *r* = −0.02, *P* = 0.9 for PCBN). Similar results were obtained for FASN methylation in the JHU cohort (*r* = −0.03, *P* = 0.6 for age and *r* = 0.03, *P* = 0.6 for preoperative PSA). FASN expression was not significantly associated with tumor Grade Group nor pathologic stage in the JHU cohort as a whole or when examined by self-identified race, with a weak association for pathologic stage (*P* = 0.04) but not Grade Group in the overall PCBN cohort (Table 1; Supplementary Table S2). FASN methylation was similarly not significantly associated with tumor Grade Group or pathologic stage in the JHU cohort (Supplementary Table S3).

**TABLE 1:** Association of FASN protein expression with clinical-pathologic variables in JHU and PCBN cohorts

	JHU			PCBN		
	*N*	Median H-score	*P* value^a^	*N*	Median H-score	*P* value^a^
**Race**						
White	194	173.5	0.02	57	213.8	0.6
Black	177	152.7		58	195.9	
**Stage**						
T2N0 or T2Nx	164	162.1	0.5	66	215.8	0.04
T3N0 or T3Nx	173	161.2		32	172.8	
N1	31	168.3		14	217.0	
**Gleason**						
<7	49	170.4	0.7	18	215.8	0.7
3+4	62	180.5		23	217.2	
4+3	163	158.1		18	190.4	
8	55	159.1		10	214.8	
9	42	165.0		43	198.7	

^a^From Kruskal–Wallis test.

**FIGURE 6:**
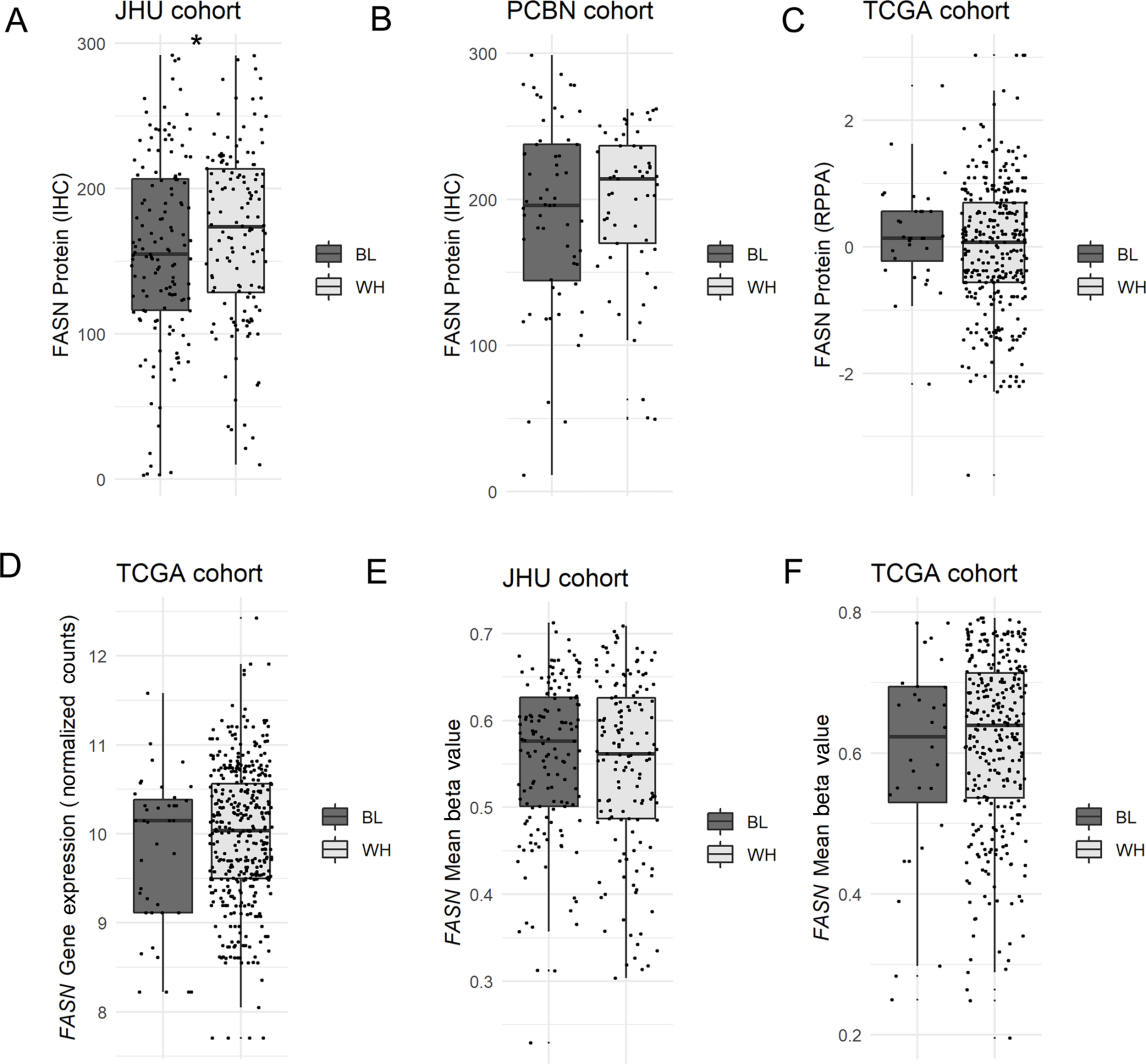
Neither FASN protein, nor *FASN* gene methylation are associated with self-identified race. **A,** FASN protein expression by race in JHU primary tumor cohort. **B,** FASN protein expression by race in PCBN primary tumor cohort. **C,** FASN protein expression by race in TCGA primary tumor cohort. **D,***FASN* gene expression by race in TCGA primary tumor cohort. **E,***FASN* gene methylation mean beta value by self-identified race in JHU primary tumor cohort. **F,***FASN* gene methylation mean beta value by self-identified race in TCGA primary tumor cohort (*, *P* < 0.05).

Surprisingly, and in contrast to two prior studies (13, 39), FASN expression was significantly higher among self-identified WH compared with BL patients in the JHU cohort (*P* = 0.02, Fig. 6A), and this trend was replicated, though not statistically significant, in the PCBN cohort (Table 1; Fig. 6B). In the TCGA cohort, there was no significant difference by race for FASN protein expression assessed by RPPA (Fig. 6C), nor for *FASN* gene expression (Fig. 6D). Similar to the results for protein and gene expression, there was no significant difference in global *FASN* gene methylation, as measured by mean beta value, when comparing the two self-identified races in the JHU (Fig. 6E) or TCGA cohorts (Fig. 6F).

Because *ERG* rearrangement is approximately half as common in BL compared with WH men and ERG status is also associated with FASN expression, we used a generalized linear regression model to estimate median difference in FASN expression by self-identified race or ERG status, adjusted by age, preoperative PSA, Grade Group, pathologic stage, and cohort. In this model, there was a significant difference in FASN expression by ERG status (*P* = 0.02 for JHU or *P* = 0.04 for PCBN) but not by race (*P* = 0.1 in JHU or *P* = 0.8 in PCBN) and there was no significant interaction between ERG status and race in either cohort (*P* = 0.9 for JHU and *P* = 0.6 for PCBN; Supplementary Table S4).

Finally, we also examined the association of FASN protein expression or methylation with risk of metastasis in the JHU and PCBN cohorts, both in univariable and multivariable models adjusted for age, preoperative PSA, Grade Group, and pathologic stage. As reported previously (8), there was no significant association of FASN as a continuous variable with metastasis in either cohort in univariable or multivariable models overall (Table 2) nor when each self-identified race was analyzed separately (Supplementary Table S5). We performed similar analyses to examine the association of global *FASN* gene methylation, as measured by mean beta value, with metastasis with similarly nonsignificant results (Supplementary Table S6). Finally, we examined the association of FASN protein expression with metastasis in the combined JHU and PCBN cohorts, stratifying by BMI because high FASN was previously shown to be associated with lethal prostate cancer specifically for obese patients (8). Using multivariable models, the HR for prostate cancer metastasis with high FASN expression was greater than 1 for patients with high BMI, but less than 1 for patients with low BMI, though FASN expression level was not statistically significant in either model (Supplementary Table S7). In a nonstratified multivariable model, FASN level was not associated with a significantly increased risk of metastasis overall, but the interaction between FASN and BMI was significant for risk of metastasis (*P* = 0.02). Taken together, these findings support prior work suggesting that FASN level may be an adverse prognostic feature specifically in overweight or obese patients.

**TABLE 2:** Cox analysis of hazard ratio (HR) for association of FASN protein expression with prostate cancer metastasis in combined cohorts.

	JHU				PCBN			
Variable	Univariable analysis		Multivariable analysis^a^		Univariable analysis		Multivariable analysis^b^	
	HR (95% CI)	*P* value	HR (95% CI)	*P* value	HR (95% CI)	*P* value	HR (95% CI)	*P* value
Average FASN (continuous)	1.003 (0.998–1.008)	0.2	1.003 (0.997–1.008)	0.3	1.006 (0.989–1.023)	0.5	1.004 (0.978–1.031)	0.8

^a^Adjusted for age, preoperative PSA, Grade Group, pathologic stage, and cohort.

^b^Adjusted for age, race, preoperative PSA, pathologic stage, and Grade Group.

## Discussion

With the exception of the liver and adipose tissue, normal cells typically express low endogenous levels of FASN and do not require *de novo* synthesis of fatty acids to supplement dietary supply. In proliferating cells, however, FASN upregulation may facilitate neoplastic lipogenesis, essential for tumorigenic cell growth, survival, and metabolism in many malignancies, including prostate cancer (40). During tumorigenesis, there is increased expression of FASN, along with increased enzymatic activity; as much as 90% of fatty acids present in tumor cells are due to *de novo* endogenous fatty acid synthesis (6). Moreover, there are compelling links between FASN expression and worse oncologic outcomes across multiple tumor types, and a significant interaction with BMI has been noted for both prostate and colon cancer (8, 41), highlighting FASN as a metabolic oncogene. Accordingly, numerous small-molecule FASN inhibitors have been developed, including Cerulenin and Orlistat, as well as, more recently, TVB-2640 and IPI-9119 (9) which are being testing in clinical trials. Developing highly validated assays to measure FASN expression as well as elucidating the mechanisms of its regulation are particularly important to aid in potential future biomarker-selected trials.

Historically, regulation of FASN expression has been relatively poorly understood. *FASN* gene amplification occurs in prostate cancer cells, correlating with protein expression (7) and germline SNPs also correlated with expression (8), suggesting potential genomic regulatory mechanisms. Transcriptionally, *FASN* is likely regulated in part by AR signaling, which has recently been found to bind to the promoter region of the gene (13) and may also stabilize the protein via upregulation of USP2a which prevents ubiquitin-mediated FASN degradation (42). Most recently, epigenetic suppression of *FASN* expression has been proposed to occur via histone deacetylation mediated by HOXB13 (15), and in preclinical studies the germline *HOXB13* G84E mutation was shown to be associated with derepression of FASN expression. In the current study, we were able to confirm this finding in human samples at the protein level. Though prostate tumors from *HOXB13* G84E carriers are frequently low grade and indolent, these results may have therapeutic implications for the rare carriers who develop metastatic disease and who may benefit from FASN inhibitors.

Consistent with our findings and the possibility of epigenetic regulation, FASN was also recently identified in a larger screen of hypomethylated and transcriptionally upregulated genes in TCGA primary prostate tumor cohort (43). Using a rigorously validated IHC assay combined with digitally quantified image analysis to evaluate FASN protein expression, we are the first to demonstrate that global *FASN* methylation level is highly inversely correlated with its protein expression. This association holds up when comparing benign and tumor cells, as well as comparing within primary or metastatic tumor cohorts, and remains significant when cases are stratified by molecular alterations associated with FASN expression, such as *ERG* gene fusions. Similar findings have been reported for numerous other genes, such as *HOXB13* (15) or genes that are hypermethylated and underexpressed in tumor compared with benign, such as HLA class I genes (44). Though these studies have proposed gene body methylation as a mechanism of gene expression regulation, this association certainly does not imply causation without detailed mechanistic experiments.

Compared with promoter methylation, gene body methylation is far more prevalent but less well characterized. Gene body methylation may work to prevent spurious transcription initiation from ectopic promoters (45), or it may ensure splicing fidelity by preventing exon skipping (46). However, it can correlate either positively or negatively with canonical gene transcription (47) because the relationship between gene body methylation and gene expression is bell-shaped, with the lowest levels of methylation observed for genes with either the highest or lowest levels of gene expression (48). This unusual finding has led some to posit that gene body methylation may be an effect, rather than a cause, of decreased transcription (48). In this model, dense nucleosome packaging in untranscribed genomic regions impedes DNA methyl transferase access to DNA. At the other extreme, in highly transcribed regions, Pol2 density may similarly impair DNA access. Further mechanistic work is required to more fully elucidate the role of gene body methylation in the regulation of FASN; however, our study suggests that at a minimum, *FASN* global methylation level is an excellent biomarker for FASN expression. In this way, *FASN* methylation level could potentially be useful in future clinical trials of FASN inhibitors because DNA methylation is often more robust to preclinical variables compared with protein biomarkers, and may even be measured accurately in circulating tumor DNA (49).

Finally, it is notable that we were unable to replicate the finding that FASN expression in prostate cancer varies by self-identified race, with higher expression in tumors from BL compared with WH patients seen in two previous studies (13, 39). Surprisingly, in two independent cohorts, we actually saw the opposite trend toward decreased FASN expression in tumors from BL patients. This paradoxical finding was due to the lower frequency of *ERG* fusions among the tumors from BL patients. ERG expression is independently associated with increased FASN expression (14), as well as increased expression of other fatty acid metabolic genes (50), in prostate cancer. After adjusting for ERG status, there was no difference by race for FASN protein expression in our grade-matched cohorts. Important differences between our study and the previous immunostaining study include the use of FASN digital quantification in our study, as well as the use of cohorts matched on most clinical pathologic parameters with adjustment for ERG status (13). Notably, at least one prior study has also suggested that *FASN* gene amplification may also be more common among prostate tumors from self-identified BL compared with WH patients (51). We were also unable to replicate this finding in our recent copy-number profiling of the JHU cohort (21), consistent with the lack of increased FASN protein expression identified in these cases.

In conclusion, we find that *FASN* gene body methylation is significantly inversely correlated with FASN expression across multiple primary and metastatic prostate cancer cohorts, and we demonstrate that prostate tumors from carriers of the germline *HOXB13* G84E mutation show increased FASN expression, consistent with recent evidence of epigenetic FASN regulation *in vitro*. However, we cannot confirm any difference in FASN expression by self-identified race. Taken together, these data may contribute to the design of future clinical trials of FASN inhibitors, providing potential biomarkers to enrich trials with patients who can derive the maximum benefit from these therapies.

## Supplementary Material

Supplementary Tables S1-S7 Supplementary Tables S1-S7

Supplementary Figure S1 Validation of FASN immunostaining assay in genetically characterized cancer cell lines.

Supplementary Figure S2 Digital quantification of FASN immunostaining in prostate tumor cells

Supplementary Figure S3 FASN gene expression is upregulated and FASN gene is hypomethylated in primary prostate tumors from the TCGA primary tumor cohort.

Supplementary Figure S4 FASN gene expression is upregulated and FASN gene is hypomethylated in primary prostate tumors from the NCI primary tumor cohort.

Supplementary Figure S5 Correlation between FASN gene expression and FASN gene methylation in the (A) JHU cohort, (B) TCGA cohort, and (C) NCI cohort.

Supplementary Figure S6 Representative whole genome bisulfite sequencing in the WCDT cohort for TMPRSS2 and KLK3.

Supplementary Figure S7 FASN gene expression and methylation is significantly associated with ERG status in TCGA primary tumor cohort.

Supplementary Figure S8 FASN gene expression is significantly associated with ERG status in NCI primary tumor cohort.
