# Supplementary material for: FASN Gene Methylation is Associated with Fatty Acid Synthase Expression and Clinical-genomic Features of Prostate Cancer
Source: Cancer Res Commun. 2024 Jan 18;4(1):152–63. doi: 10.1158/2767-9764.CRC-23-0248 (PMC10795515; doi:10.1158/2767-9764.CRC-23-0248)
Supplement: Supplementary Tables S1-S7 [file crc-23-0248-s01.docx]

|  | **cg17928916** | **cg23890876** | **cg03407524** | **cg06463097** | **cg04029738** | **cg17462356** | **cg14524553** |
| --- | --- | --- | --- | --- | --- | --- | --- |
| **Spearman's Rho** | -0.12892681 | -0.26081624 | -0.13465162 | -0.31839785 | -0.27099442 | -0.0281343 | -0.18681982 |
|  | **cg15948410** | **cg05953751** | **cg03386722** | **cg15818577** | **cg16699850** | **cg25825740** | **cg19250315** |
| **Spearman's Rho** | -0.23972432 | -0.07541616 | -0.13427662 | -0.21737019 | -0.25206832 | -0.14023944 | -0.27698121 |
|  | **cg01565083** | **cg11950105** | **cg06234966** | **cg23244421** | **cg24715260** | **cg27460534** | **cg06259406** |
| **Spearman's Rho** | -0.05546211 | -0.28928183 | -0.23239337 | -0.11567072 | 0.006426278 | -0.21914699 | -0.3092513 |
|  | **cg03693434** | **cg15729979** | **cg01291508** | **cg13804838** | **cg06906087** | **cg26994671** | **cg06875616** |
| **Spearman's Rho** | -0.28106791 | -0.23553831 | -0.17985132 | -0.20298968 | -0.1574304 | -0.24373282 | -0.31082947 |
|  | **cg04683330** | **cg22158723** | **cg18009376** | **cg25503540** | **cg05396584** | **cg19183833** | **cg20276947** |
| **Spearman's Rho** | -0.31322498 | -0.35285969 | -0.06578274 | -0.16988742 | -0.29336853 | -0.25642842 | -0.02741456 |
|  | **cg25423135** | **cg17581200** | **cg25068915** | **cg04443493** | **cg02289168** | **cg17512993** | **cg11958594** |
| **Spearman's Rho** | -0.09131262 | -0.21825602 | -0.21263338 | -0.15596581 | -0.28792454 | -0.23528831 | -0.0505672 |
|  | **cg27485530** | **cg20592144** | **cg20482334** | **cg13823188** | **cg14797580** | **cg07247860** | **cg05157970** |
| **Spearman's Rho** | -0.26816398 | -0.29346385 | -0.1768428 | -0.26244317 | -0.06047973 | -0.25244674 | -0.27730712 |
|  | **cg08051977** | **cg22622425** | **cg09799307** | **cg14521508** | **cg05806884** | **cg09819958** | **cg02836807** |
| **Spearman's Rho** | -0.24972532 | -0.23312396 | -0.14619426 | -0.06102824 | -0.15090766 | -0.22348483 | -0.1405448 |

**Supplementary Table S1A: Spearman correlations for *FASN* CpG methylation probe beta values versus FASN protein expression by immunostaining in JHU cohort**

**Supplementary Table S1B: Spearman correlations for *FASN* CpG methylation probe beta values versus FASN protein expression by RPPA in TCGA cohort**

|  | **cg01565083** | **cg03407524** | **cg04029738** | **cg04683330** | **cg05396584** | **cg06234966** |  |
| --- | --- | --- | --- | --- | --- | --- | --- |
| **Spearman's Rho** | -0.34116932 | -0.24152325 | -0.37192769 | -0.39592136 | -0.39344306 | -0.32441615 |  |
|  | **cg02836807** | **cg03386722** | **cg03693434** | **cg05157970** | **cg05384347** | **cg05953751** |  |
| **Spearman's Rho** | -0.34807112 | -0.01134164 | -0.37990648 | -0.38051457 | -0.3570849 | -0.2012684 |  |
|  | **cg19183833** | **cg20276947** | **cg20482334** | **cg20592144** | **cg22158723** | **cg23244421** |  |
| **Spearman's Rho** | -0.35090761 | -0.1170489 | -0.37024469 | -0.37479538 | -0.3618972 | -0.0389359 |  |
|  | **cg14797580** | **cg15729979** | **cg15818577** | **cg15948410** | **cg16699850** | **cg17512993** |  |
| **Spearman's Rho** | -0.22800737 | -0.39022796 | -0.39862465 | -0.36532289 | -0.33716583 | -0.38307177 |  |
|  | **cg26994671** | **cg18009376** | **cg17928916** | **cg14521508** | **cg13804838** | **cg02289168** |  |
| **Spearman's Rho** | -0.31928045 | -0.05609828 | -0.34882261 | -0.04898799 | -0.24585854 | -0.34924891 |  |
|  | **cg06259406** | **cg06463097** | **cg11950105** | **cg13823188** | **cg14524553** | **cg25825740** |  |
| **Spearman's Rho** | -0.3605985 | -0.39477056 | -0.37802788 | -0.3641346 | -0.31861025 | -0.25139864 |  |
|  | **cg06875616** | **cg06906087** | **cg08051977** | **cg09799307** | **cg09819958** | **cg17462356** |  |
| **Spearman's Rho** | -0.37673428 | -0.23155876 | -0.39062366 | -0.38064747 | -0.34780262 | -0.03110681 |  |
|  | **cg23890876** | **cg24715260** | **cg25503540** | **cg27460534** | **cg27485530** | **cg11958594** |  |
| **Spearman's Rho** | -0.40230595 | -0.00966734 | -0.31823615 | -0.39916345 | -0.32538514 | -0.02455032 |  |
|  | **cg17581200** | **cg19250315** | **cg22622425** | **cg23355636** | **cg25068915** | **cg01291508** | **cg25423135** |
| **Spearman's Rho** | -0.34236212 | -0.32381285 | -0.38830767 | -0.38012188 | -0.32970484 | -0.34649012 | -0.22247267 |

|  |  | **Supplementary Table S2. Association of FASN protein expression with clinical-pathologic variables by self-identified race in JHU and PCBN cohorts** | | | | | | | | | | | | | | | | |  |
| --- | --- | --- | --- | --- | --- | --- | --- | --- | --- | --- | --- | --- | --- | --- | --- | --- | --- | --- | --- |
|  | |  | | | JHU | | | | |  |  | | PCBN | | | | |  | |
|  | |  | White | | | |  | Black | |  |  | White | | |  | Black | |  | |
|  | | N | Median | | | P Value* | N | Median | P Value* |  | N | Median | | P Value* | N | Median | P Value* |  | |
| Stage | |  |  | | |  |  |  |  |  |  |  | |  |  |  |  |  | |
| T2 | | 88 | | 181.0 | | 0.7 | 76 | 151.6 | 0.5 |  | 34 | 215.8 | | 0.1 | 32 | 209.0 | 0.3 |  | |
| T3/T4 | | 89 | | 169.3 | |  | 84 | 152.3 |  |  | 16 | 172.8 | |  | 16 | 175.0 |  |  | |
| N1 | | 17 | | 175.2 | |  | 14 | 167.1 |  |  | 7 | 219.1 | |  | 7 | 195.7 |  |  | |
|  | |  |  | | |  |  |  |  |  |  |  | |  |  |  |  |  | |
| Gleason | |  |  | | |  |  |  |  |  |  |  | |  |  |  |  |  | |
| <7 | | 26 | 187.7 | | | 0.1 | 23 | 158.6 | 0.9 |  | 9 | 215.8 | | 0.8 | 9 | 200.7 | 0.6 |  | |
| 3+4 | | 33 | 192.7 | | |  | 29 | 147.2 |  |  | 12 | 219.1 | |  | 11 | 217.2 |  |  | |
| 4+3 | | 84 | 165.5 | | |  | 79 | 154.1 |  |  | 10 | 212.5 | |  | 8 | 150.9 |  |  | |
| 8 | | 28 | 174.8 | | |  | 27 | 147.8 |  |  | 5 | 213.8 | |  | 5 | 231.4 |  |  | |
| 9 | | 23 | 165.7 | | |  | 19 | 164.3 |  |  | 21 | 203.3 | |  | 22 | 192.5 |  |  | |
|  | |  |  | | |  |  |  |  |  |  |  | |  |  |  |  |  | |
|  |  | *, from Kruskal-Wallis Test | | | | | | | | | | | | | | | | |  |

| **Supplementary Table S3. Comparisons of *FASN* Mean Beta Value in JHU Cohort** | | | | | | | | | | | |
| --- | --- | --- | --- | --- | --- | --- | --- | --- | --- | --- | --- |
|  | All | | |  | White | | |  | Black | | |
|  | N | Median | P Value* |  | N | Median | P Value* |  | N | Median | P Value* |
| Stage |  |  |  |  |  |  |  |  |  |  |  |
| T2N0 or T2Nx | 128 | 0.551 | 0.1 |  | 63 | 0.559 | 0.5 |  | 65 | 0.538 | 0.1 |
| T3N0 or T3Nx | 138 | 0.577 |  |  | 70 | 0.567 |  |  | 68 | 0.591 |  |
| N1 | 23 | 0.590 |  |  | 12 | 0.575 |  |  | 11 | 0.596 |  |
|  |  |  |  |  |  |  |  |  |  |  |  |
| Gleason |  |  |  |  |  |  |  |  |  |  |  |
| <7 | 38 | 0.546 | 0.8 |  | 18 | 0.559 | 0.9 |  | 20 | 0.542 | 0.5 |
| 3+4 | 39 | 0.586 |  |  | 20 | 0.561 |  |  | 19 | 0.599 |  |
| 4+3 | 136 | 0.565 |  |  | 70 | 0.561 |  |  | 66 | 0.571 |  |
| 8 | 44 | 0.579 |  |  | 21 | 0.578 |  |  | 23 | 0.581 |  |
| 9 | 32 | 0.577 |  |  | 16 | 0.562 |  |  | 16 | 0.587 |  |
|  |  |  |  |  |  |  |  |  |  |  |  |
|  |  |  |  |  |  |  |  |  |  |  |  |
| *, from Kruskal-Wallis Test | | | | | | | | | | | |

|  | **Supplementary Table S4. Difference in Median FASN H-score by cohort** | | | | | |
| --- | --- | --- | --- | --- | --- | --- |
|  |  | | | |  |  |
|  |  | JHU | | PCBN |  |  |
|  |  | Median Difference  (95% CI) | *p* value | Median Difference  (95% CI) | *p* value |  |
| Race |  |  |  |  |  |  |
| White vs  Black |  | 18.6  (-0.9-38.0) | 0.1 | 3.5  (-24.9 -32.0) | 0.8 |  |
| ERG |  |  |  |  |  |  |
| Positive vs  Negative |  | 29.1  (4.7-53.6) | 0.02 | 40.0  (1.1 - 78.9) | 0.04 |  |
| P-interaction |  |  | 0.9 |  | 0.6 |  |
|  | *, a generalized linear regression model with race, ERG status and the interaction between race and ERG status, adjusted by age, stage, Grade Group, preoperative PSA and cohort. | | | | | |

| **Supplementary Table S5A.** **Cox Analysis of Hazard Ratio (HR) of Association of FASN Protein Expression with Prostate Cancer Metastasis in JHU Cohort** | | | | | | | | | | |
| --- | --- | --- | --- | --- | --- | --- | --- | --- | --- | --- |
|  | White | | | |  | Black | | | |  |
| Variable | Univariable Analysis | | Multivariable Analysis* | |  | Univariable Analysis | | Multivariable Analysis* | |  |
|  | HR  (95% CI) | *p* value | HR  (95% CI) | HR  (95% CI) | *p* value | HR  (95% CI) | HR  (95% CI) | *p* value | HR  (95% CI) |  |
| Average FASN H-score (continuous) | 1.004  (0.997-1.011) | 0.3 | 1.004  (0.997-1.011) | 1.004  (0.997-1.011) | 0.3 | 1.004  (0.997-1.011) | 1.004  (0.997-1.011) | 0.3 | 1.004  (0.997-1.011) |  |
| *, adjusted for age, pre-operative PSA, Grade Group, pathologic stage, and cohort. | | | | | | | | | | |

| **Supplementary Table S5B.** **Cox Analysis of Hazard Ratio (HR) of Association of FASN Protein Expression with Prostate Cancer Metastasis in PCBN Cohort** | | | | | | | | | | | |
| --- | --- | --- | --- | --- | --- | --- | --- | --- | --- | --- | --- |
|  |  | White | | | |  | Black | | | |  |
| Variable |  | Univariable Analysis | | Multivariable Analysis* | |  | Univariable Analysis | | Multivariable Analysis* | |  |
|  |  | HR  (95% CI) | *p* value | HR  (95% CI) | *p* value |  | HR  (95% CI) | *p* value | HR  (95% CI) | *p* value |  |
| Average FASN H-score (continuous) |  | 0.998  (0.978-1.020) | 0.9 | 0.969  (0.899-1.043) | 0.4 |  | 1.011  (0.986-1.038) | 0.4 | - |  |  |
| *, adjusted for age, pre-operative PSA, Grade Group, and pathologic stage. | | | | | | | | | | | |

| **Supplementary Table S6. Cox Analysis of Hazard Ratio (HR) of Association of *FASN* Gene Methylation with Prostate Cancer Metastasis in JHU Cohort** | | | | | | | | | | | | | | |
| --- | --- | --- | --- | --- | --- | --- | --- | --- | --- | --- | --- | --- | --- | --- |
|  | All | | | |  | White | | | |  | Black | | | |
| Variable | Univariable Analysis | | Multivariable Analysis* | |  | Univariable Analysis | | Multivariable Analysis** | |  | Univariable Analysis | | Multivariable Analysis** | |
|  | HR  (95% CI) | *p* value | HR  (95% CI) | *p* value |  | HR  (95% CI) | *p* value | HR  (95% CI) | *p* value |  | HR  (95% CI) | *p* value | HR  (95% CI) | *p* value |
| FASN Methylation Measurement (continuous) | 1.876 (0.044 -79.657) | 0.7 | 0.474 (0.010 -22.838) | 0.7 |  | 0.507 (0.005 -52.570) | 0.8 | 0.238 (0.002 -23.281) | 0.5 |  | 17.629 (0.022 -14063.56) | 0.4 | 1.228  (0.000 -5126.709) | 0.9 |
| *, adjusted for age, preoperative PSA, pathologic stage, Grade Group | | | | | | | | | | | | | | |

| **Supplementary Table S7. Adjusted Hazard Ratios (HRs) of Prostate Cancer Metastasis in Combined JHU and PCBN Cohorts by FASN protein Expression and Body Mass Index (BMI)** | | | | | | | | | | | |
| --- | --- | --- | --- | --- | --- | --- | --- | --- | --- | --- | --- |
|  | All ** | | |  | BMI<25 *** | | |  | BMI>=25 *** | | |
|  | N  (Cases/Controls) | HR  (95% CI) | *p* value |  | N  (Cases/Controls) | HR  (95% CI) | *p* value |  | N  (Cases/Controls) | HR  (95% CI) | *p* value |
| Average FASN Low | 11/87 | Ref | - |  | 4/15 | Ref | - |  | 7/72 | Ref | - |
| Average FASN High | 23/175 | 1.058  (0.484 -2.314) | 0.9 |  | 4/42 | 0.988  (0.087 -11.171) | 0.9 |  | 19/133 | 1.761  (0.671 -4.620) | 0.3 |
| P interaction between FASN (low vs high) and BMI (<25 vs >=25) |  |  | 0.02 |  |  |  |  |  |  |  |  |
|  |  |  |  |  |  |  |  |  |  |  |  |
| *, participants from JHU and PCBN cohorts were combined. Tertiles of average FASN or maximum FASN were created using cohort-specific cut points. The top two tertiles were combined as average or maximum FASN high, and the bottom tertile was average or maximum FASN low.  **, adjusted for age, race, stage, gleason, preoperative PSA, cohort, and BMI (continuous).  ***, adjusted for age, race, stage, gleason, preoperative PSA, and cohort. | | | | | | | | | | | |
