# Supplementary material for: FASN Gene Methylation is Associated with Fatty Acid Synthase Expression and Clinical-genomic Features of Prostate Cancer
Source: Cancer Res Commun. 2024 Jan 18;4(1):152–63. doi: 10.1158/2767-9764.CRC-23-0248 (PMC10795515; doi:10.1158/2767-9764.CRC-23-0248)
Supplement: Supplementary Figure S1 — Validation of FASN immunostaining assay in genetically characterized cancer cell lines. [file crc-23-0248-s02.pdf]

Supplementary Figure S1

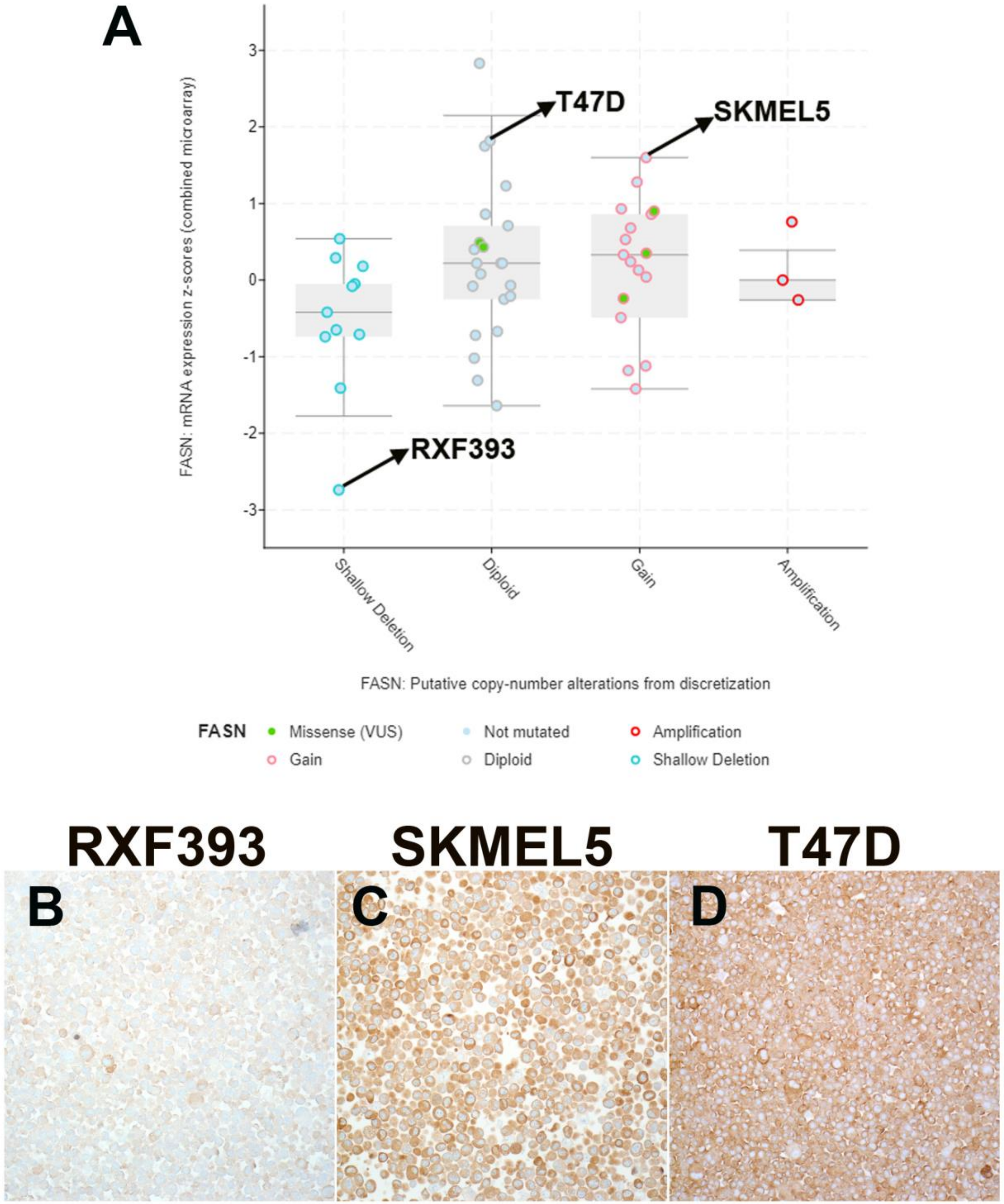

Supplementary Figure S1: Validation of FASN immunostaining assay in genetically characterized cancer cell lines. A. Transcript levels for *FASN* across the NCI-60 cell line panel shows RXF393 (renal cell carcinoma cell line) has a shallow deletion involving the *FASN* gene, with concomitantly low *FASN* gene expression, while T47D (breast cancer cell line) and SKMEL5 (melanoma cell line) are diploid or have a gain in the *FASN* gene leading to high expression at the mRNA level. B. FASN protein is expressed at low levels by immunostaining in RXF393, consistent with the low mRNA level. C. FASN protein is expressed at high levels by immunostaining in SKMEL5, consistent with the high mRNA level. D. FASN protein is expressed at high levels by immunostaining in T47D, consistent with the high mRNA level.
