# Supplementary material for: FASN Gene Methylation is Associated with Fatty Acid Synthase Expression and Clinical-genomic Features of Prostate Cancer
Source: Cancer Res Commun. 2024 Jan 18;4(1):152–63. doi: 10.1158/2767-9764.CRC-23-0248 (PMC10795515; doi:10.1158/2767-9764.CRC-23-0248)
Supplement: Supplementary Figure S2 — Digital quantification of FASN immunostaining in prostate tumor cells [file crc-23-0248-s03.pdf]

Supplementary Figure S2

A

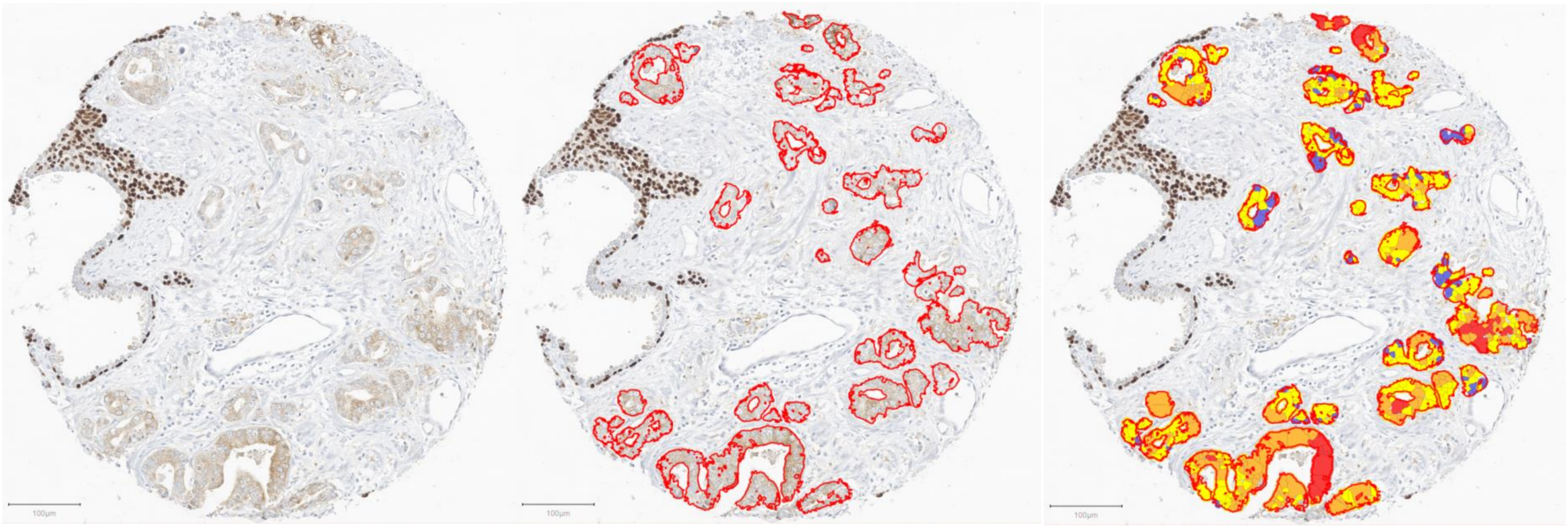

B

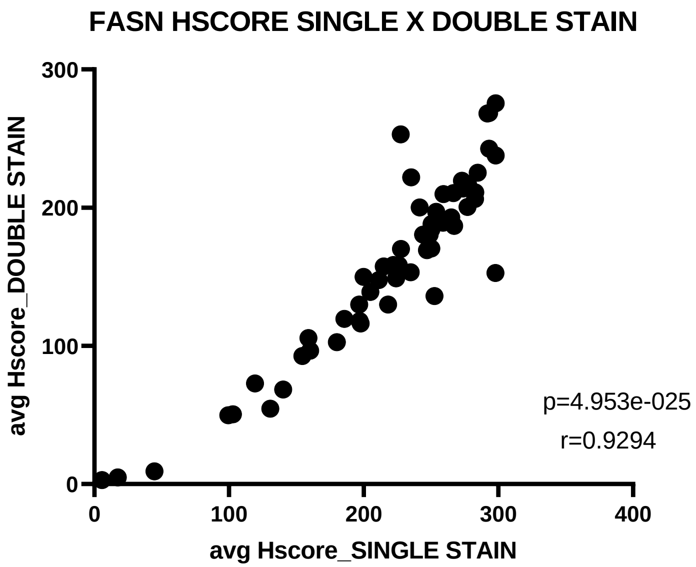

Supp Figure S2. Digital quantification of FASN immunostaining in prostate tumor cells. A. QuPath algorithms were used to identify tumor epithelium within each core (red outlines in middle panel) and benign glands were excluded based on intact staining for p63-positive basal cells (brown nuclear staining) in a double-stained image. QuPath intensity scores were assigned to each segmented cell (blue=0+, yellow=1+, orange=2+, red=3+ and the overall H-score for each core determined. B. The correlation between the H-score assessed on the double-stained slide (p63/FASN) and the H-score assessed on the single-stained slide (FASN only) was almost perfect across 60 cases, suggesting no effect of double-staining with p63 on FASN protein quantification, thus all scoring in this study was performed on the double-stained slide to enable distinction of tumor from benign glands.
