# Supplementary material for: FASN Gene Methylation is Associated with Fatty Acid Synthase Expression and Clinical-genomic Features of Prostate Cancer
Source: Cancer Res Commun. 2024 Jan 18;4(1):152–63. doi: 10.1158/2767-9764.CRC-23-0248 (PMC10795515; doi:10.1158/2767-9764.CRC-23-0248)
Supplement: Supplementary Figure S3 — FASN gene expression is upregulated and FASN gene is hypomethylated in primary prostate tumors from the TCGA primary tumor cohort. [file crc-23-0248-s04.pdf]

Supplementary Figure S3

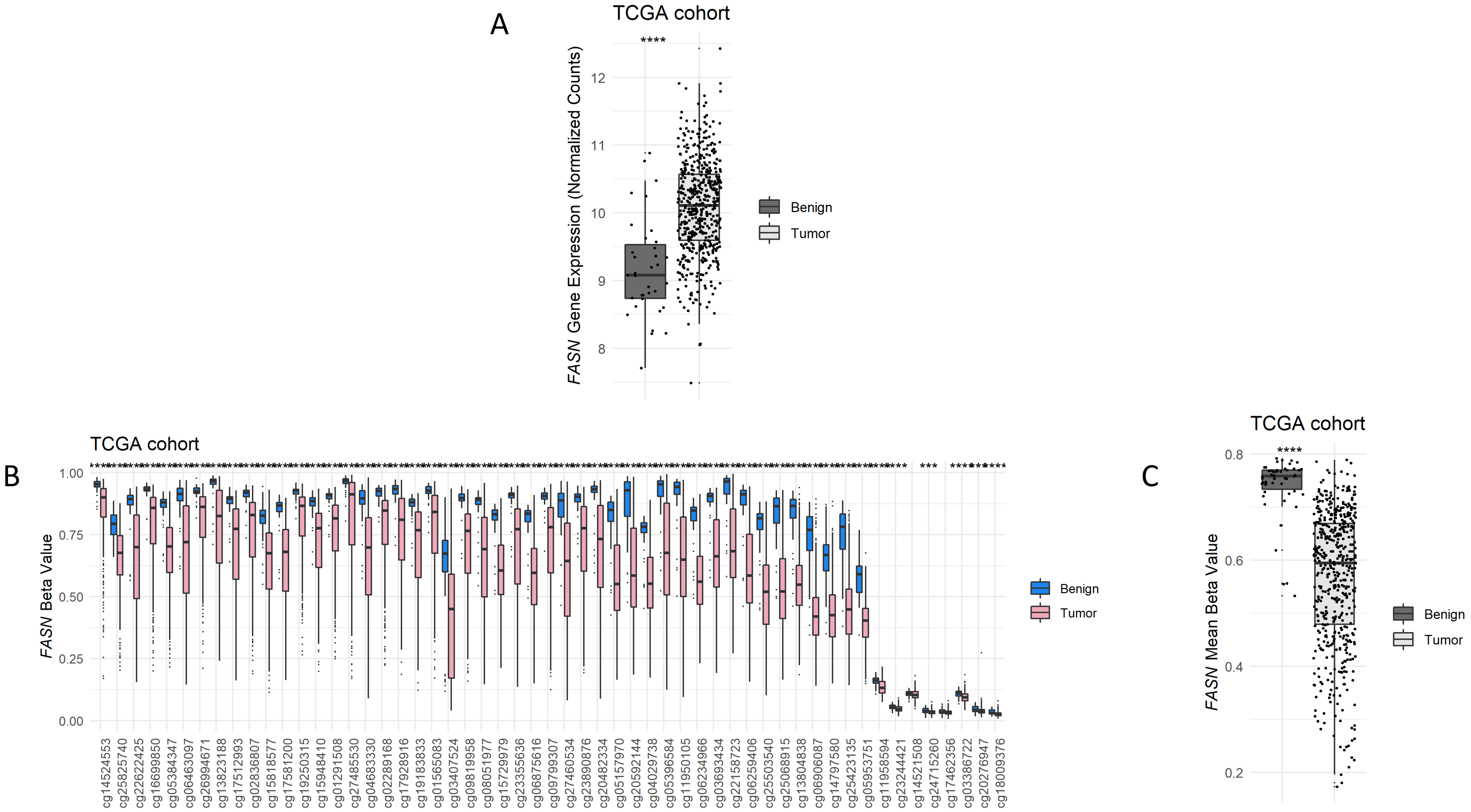

Supplementary Figure S3. *FASN* gene expression is upregulated and *FASN* gene is hypomethylated in primary prostate tumors from the TCGA primary tumor cohort. A. *FASN* gene expression in benign prostate glands versus tumor glands. B. *FASN* gene methylation probe beta values for benign tissue versus tumor tissue. C. Mean *FASN* gene methylation probe beta value for benign tissue versus tumor tissue.
