# Supplementary material for: FASN Gene Methylation is Associated with Fatty Acid Synthase Expression and Clinical-genomic Features of Prostate Cancer
Source: Cancer Res Commun. 2024 Jan 18;4(1):152–63. doi: 10.1158/2767-9764.CRC-23-0248 (PMC10795515; doi:10.1158/2767-9764.CRC-23-0248)
Supplement: Supplementary Figure S5 — Correlation between FASN gene expression and FASN gene methylation in the (A) JHU cohort, (B) TCGA cohort, and (C) NCI cohort. [file crc-23-0248-s06.pdf]

Supplementary Figure S5

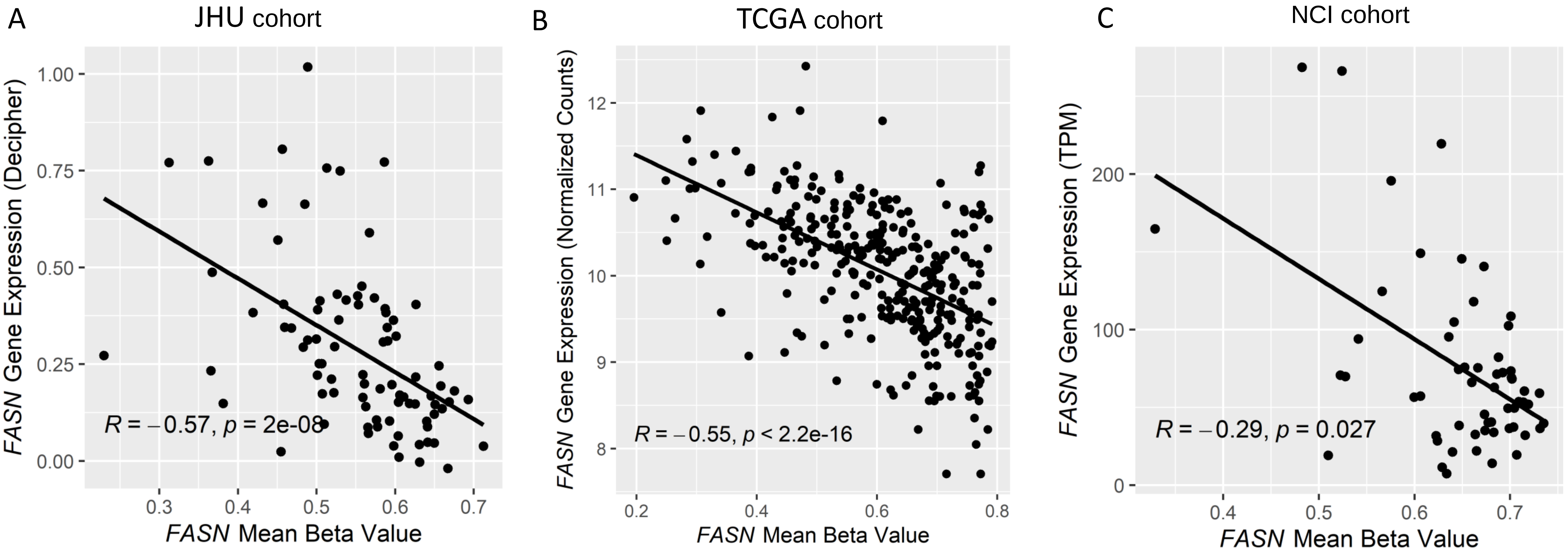

Supplementary Figure S5. Correlation between *FASN* gene expression and *FASN* gene methylation in the (A) JHU cohort, (B) TCGA cohort, and (C) NCI cohort.
