# Supplementary material for: FASN Gene Methylation is Associated with Fatty Acid Synthase Expression and Clinical-genomic Features of Prostate Cancer
Source: Cancer Res Commun. 2024 Jan 18;4(1):152–63. doi: 10.1158/2767-9764.CRC-23-0248 (PMC10795515; doi:10.1158/2767-9764.CRC-23-0248)
Supplement: Supplementary Figure S6 — Representative whole genome bisulfite sequencing in the WCDT cohort for TMPRSS2 and KLK3. [file crc-23-0248-s07.pdf]

Supplementary Figure S6

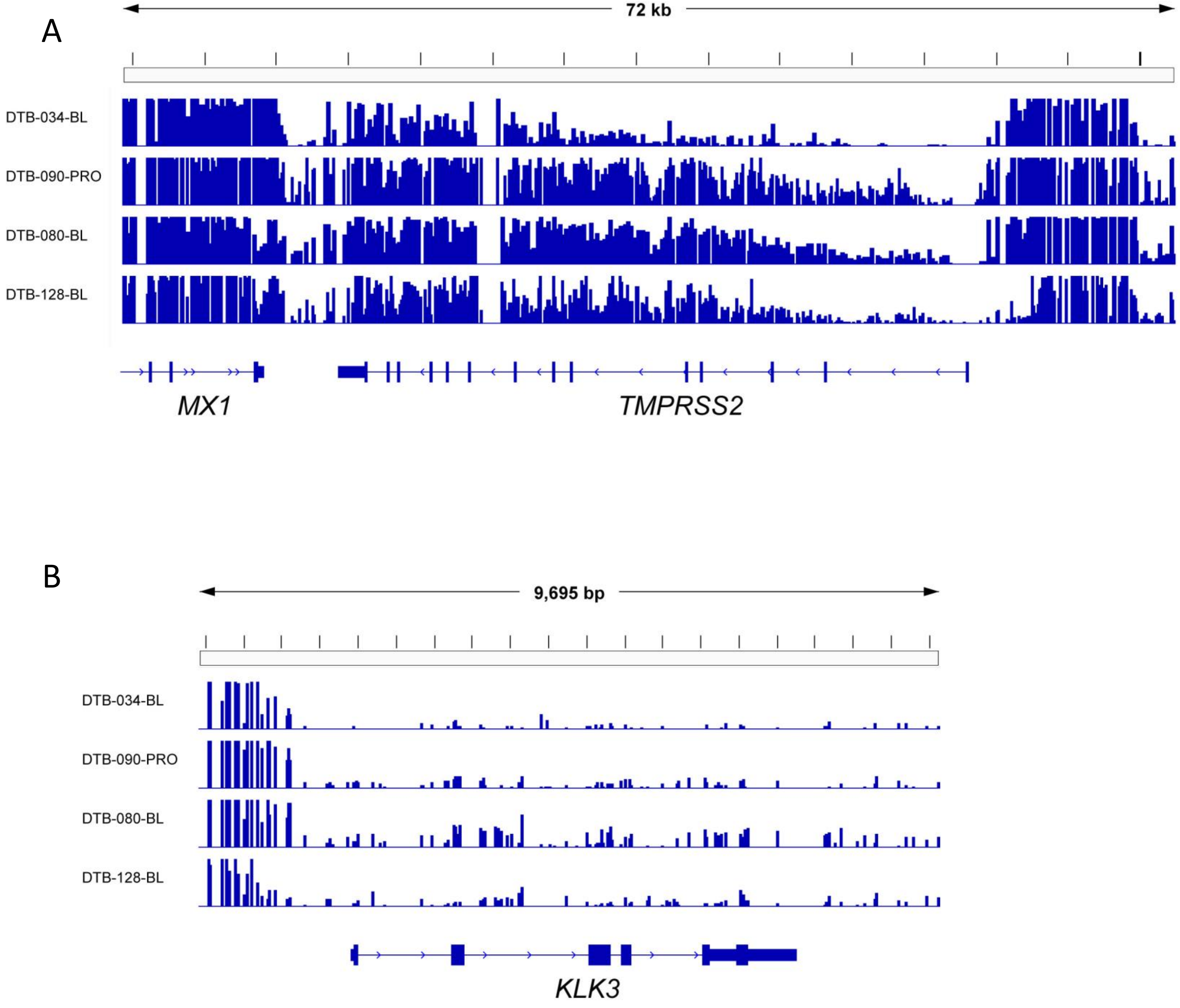

Supplementary Figure S6. Representative whole genome bisulfite sequencing in the WCDT cohort for *TMPRSS2* and *KLK3*.
