# Supplementary material for: FASN Gene Methylation is Associated with Fatty Acid Synthase Expression and Clinical-genomic Features of Prostate Cancer
Source: Cancer Res Commun. 2024 Jan 18;4(1):152–63. doi: 10.1158/2767-9764.CRC-23-0248 (PMC10795515; doi:10.1158/2767-9764.CRC-23-0248)
Supplement: Supplementary Figure S7 — FASN gene expression and methylation is significantly associated with ERG status in TCGA primary tumor cohort. [file crc-23-0248-s08.pdf]

Supplementary Figure S7

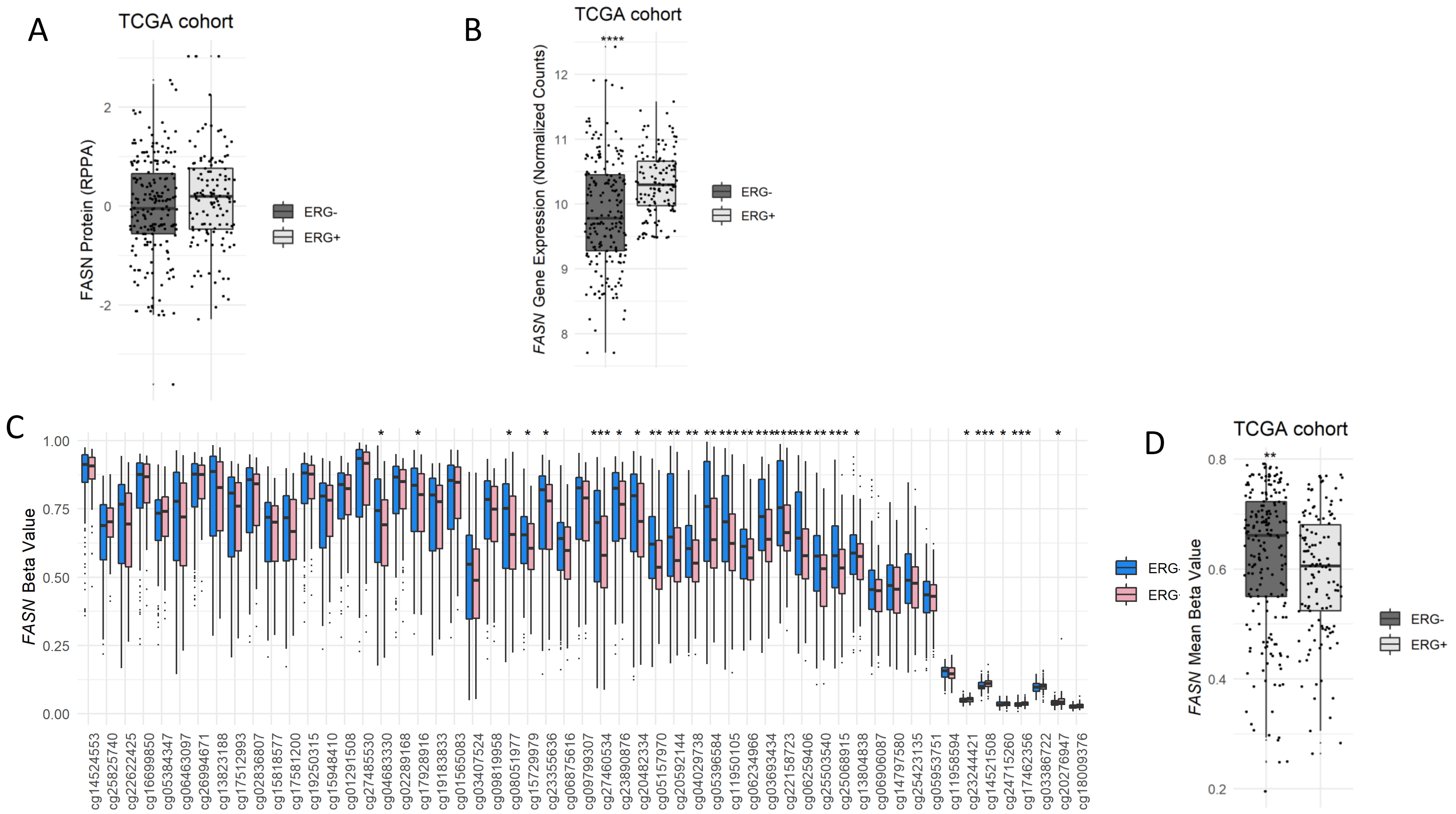

Supplementary Figure S7. *FASN* gene expression and methylation is significantly associated with *ERG* status in TCGA primary tumor cohort. A. Protein expression for FASN (via RPPA) by *ERG* fusion status. B. RNA expression for FASN (via RNAseq) by *ERG* fusion status. C. *FASN* gene methylation probe beta value by *ERG* fusion status. D. *FASN* gene methylation mean beta value by ERG status.
