# Supplementary material for: FASN Gene Methylation is Associated with Fatty Acid Synthase Expression and Clinical-genomic Features of Prostate Cancer
Source: Cancer Res Commun. 2024 Jan 18;4(1):152–63. doi: 10.1158/2767-9764.CRC-23-0248 (PMC10795515; doi:10.1158/2767-9764.CRC-23-0248)
Supplement: Supplementary Figure S8 — FASN gene expression is significantly associated with ERG status in NCI primary tumor cohort. [file crc-23-0248-s09.pdf]

Supplementary Figure S8

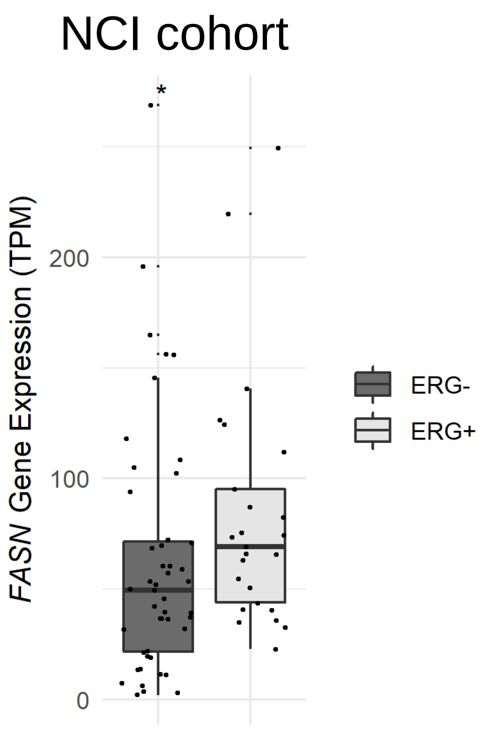

Supplementary Figure S8. ***FASN*** **gene expression is significantly associated with** ***ERG*** **status in NCI primary tumor cohort.**
